# High incidence of *Aggregatibacter actinomycetemcomitans* infection in patients with cerebral infarction and diabetic renal failure: a cross-sectional study

**DOI:** 10.1186/1471-2334-13-557

**Published:** 2013-11-24

**Authors:** Minoru Murakami, Jun-ichi Suzuki, Satoshi Yamazaki, Masaya Ikezoe, Rintaro Matsushima, Norihiko Ashigaki, Norio Aoyama, Naho Kobayashi, Kouji Wakayama, Hiroshi Akazawa, Issei Komuro, Yuichi Izumi, Mitsuaki Isobe

**Affiliations:** 1 Department of Nephrology, Saku Central Hospital, Nagano, Japan; 2 Department of Advanced Clinical Science and Therapeutics, University of Tokyo, Tokyo, Japan; 3 Department of Dentistry and Oral Surgery, Saku Central Hospital, Nagano, Japan; 4 Department of Periodontology, Tokyo Medical and Dental University, Tokyo, Japan; 5 Department of Cardiovascular Medicine, University of Tokyo, Tokyo, Japan; 6 Department of Cardiovascular Medicine, Tokyo Medical and Dental University, Tokyo, Japan

**Keywords:** Periodontitis, *Aggregatibacter actinomycetemcomitans*, Diabetic nephropathy, Cerebral infarction

## Abstract

**Background:**

Recent epidemiological studies suggest that periodontitis is a major risk factor for renal failure and cerebral infarction. The aim of this study was to evaluate the association among periodontitis, renal failure, and cerebral infarction, focusing on microbiological and immunological features.

**Methods:**

Twenty-one patients treated with hemodialysis (HD) were enrolled in this study. They were 8 with diabetic nephropathy and 13 with non-diabetic nephropathy. Blood examination, periodontal examination, brain magnetic resonance image (MRI), and dental radiography were performed on all patients. Subgingival plaque, saliva, and blood samples were analyzed for the periodontal pathogens, *Aggregatibacter actinomycetemcomitans* (*A. actinomycetemcomitans*), *Porphyromonas gingivalis* (*P. gingivalis*), and *Prevotella intermedia* (*P. intermedia*) using quantitative real-time polymerase chain reaction (qRT-PCR) and enzyme-linked immunosorbent assay (ELISA).

**Results:**

We found that the patients with diabetic nephropathy had more *A. actinomycetemcomitans* compared with non-diabetic nephropathy (P = 0.038) in dental plaque. Furthermore, the patients with diabetic nephropathy showed a significantly higher incidence of cerebral infarction compared with those with non-diabetic nephropathy (P = 0.029). Clinical oral and radiographic scores tended to be higher among patients in the diabetic nephropathy group than in the non-diabetic nephropathy group.

**Conclusions:**

Periodontal pathogens, particularly *A. actinomycetemcomitans*, may play a role, at least a part, in the development of cerebral infarction in Japanese HD patients with diabetic nephropathy.

## Background

Chronic kidney disease (CKD) is a growing public health problem that is associated with an increased risk of cardiovascular disease and mortality [1]. Reduced kidney function is associated with cardiovascular events, even when dysfunction is mild. The vascular changes in CKD patients consist not only in atherosclerosis but also in arteriosclerosis associated with both medial and intimal vascular calcification [2]. CKD is caused by a progressive and irreversible decline in the number of functioning nephrons. The patients develop end-stage renal disease (ESRD) once the damage passes the point of compensation. Therefore, hemodialysis (HD) treatment and kidney transplantation are life-saving medical procedures in these patients.

Recently, some studies demonstrated a high prevalence of periodontitis in individuals with all stages of CKD [3,4]. Periodontitis, one of the most common infections in humans, is caused by subgingival infection with predominantly gram-negative anaerobic bacteria in disease susceptible individuals. Because, this disease contributes to systemic inflammation [5], the periodontal treatment markedly reduces systemic inflammation.

Recent evidence shows that chronic inflammation may cause protein-energy malnutrition and progressive atherosclerosis in HD patients [6]. Chronic periodontal inflammation may also contribute to the chronic systemic inflammatory burden associated with CKD [7]. Thus, periodontopathic bacteria may play a key role in the progression of CKD.

Periodontal disease is known as an independent risk factor for cerebral ischemia [8]. CKD is associated with a high prevalence of stroke [9], and the presence of silent cerebral infarction increased markedly as estimated glomerular filtration rate decreased [10]. However, there was no study to clarify the pathophysiological relationship between chronic periodontitis and cerebral infarction in patients with renal failure.

Therefore, the purpose of the present study was to investigate the association among chronic periodontitis, cerebral infarction and cause of ESRD within Japanese HD patients, focusing on the microbiological and immunological features of this disease.

## Methods

### Study population

We conducted the present study of all HD patients who admitted to the hemodialysis unit at Saku Central Hospital, Nagano, Japan. Exclusion criteria included (i) known systemic diseases, (ii) history and/or presence of other infections, (iii) systemic antibiotic, immunosuppressive or periodontal treatment in the preceding 6 months prior to the sample collection, and (iv) disagreement with the present study. Informed consent was obtained from each subject after providing them with verbal and written explanations of the nature of the study. The study was approved by the Ethics Committees of Saku Central Hospital, University of Tokyo (ID 2947) and Tokyo Medical and Dental University (ID 546). Data of age, gender, height, dry weight, smoking status, cause of ESRD, dialysis prescription, and the use of drugs, including statins, antihypertensives, anticoagulants, and antiplatelets were collected from the medical records.

### Periodontal examination

This cross-sectional study was conducted between November and December 2011. Periodontal examination was performed by one experienced dentist who was masked to the clinical systemic findings of these patients. Full-mouth clinical measurements including probing pocket depth (PPD), clinical attachment level (CAL), and bleeding on probing (BOP) were recorded at 6 sites on each tooth using a manual probe (PCP-UNC 15, Hu-Friedy Manufacturing Co., Chicago, IL, USA). Oral specimens (subgingival plaque and saliva) were taken at the same time. A full-mouth set of 10 periapical radiograms was also obtained from each patient using the isometric method. Alveolar bone loss was measured using the 10 dental X-ray films. Patients underwent a standard phase of nonsurgical periodontal treatment.

### Sample collection and preparation

Laboratory data were taken from all subjects within a few days of the clinical examination during stable outpatient HD sessions. Blood samples were drawn from the arterial end of the vascular access immediately before initiation of HD, then stored at -70°C until assay. All subjects underwent a complete blood count, blood chemistry analysis, and several measures of lipid metabolism including total cholesterol (TC), triglycerides (TG), and high- and low-density lipoprotein (HDL, LDL). The serum levels of high-sensitivity C-reactive protein (hs-CRP) were also measured. Whole blood was subjected to microbiological analysis. Blood samples were also subjected to determine the specific serum IgG antibody responses to the periodontal pathogens tested. Subgingival plaque and saliva samples were collected during a periodontal examination. Subgingival plaque samples were collected from the deepest pockets in each quadrant and pooled for microbiological analysis. After supragingival debridement, subgingival plaque was collected by inserting a sterile paper point (No. 30) into the pocket until resistance was felt and was kept in place for 30 seconds. Paper points with plaque samples were transferred to a sterile vial and unstimulated saliva (500 μL) was also collected from each patient in a sterile tube. All samples were kept in a freezer at -80°C until used for the extraction of bacterial DNA. Dialysis clearance of urea was expressed as Kt/V_urea_, according to Daugirdas [11] in HD. The characteristics of the subjects, including age, sex, smoking status, and biological, hematologic, and dialysis-related data, are listed in Table 1.

**Table 1:** Baseline characteristics of the study populations

	**DM (n=8)**	**Non-DM (n=13)**	P value*
Characteristics			
Age (y)	63.0 (57.2, 68.5)	64.0 (45.5, 74.5)	0.913
Height (cm)	166.8 (160.0, 171.3)	167.0 (156.6, 169.4)	0.772
Dry weight (kg)	68.4 (58.4, 81.1)	57.0 (53.8, 65.0)	0.051
Female sex	2(25.0)	3 (23.1)	0.920
Smoking	1(12.5)	2 (15.4)	0.854
Drugs			
Antihypertensives	7(87.5)	13 (100)	0.192
Antiplatelets	5(62.5)	5 (38.5)	0.284
Anticoagulants	1(12.5)	3 (23.1)	0.549
Statins	3(37.5)	3 (23.1)	0.477
Biochemical data			
TP (g/dL)	6.9 (6.7, 7.3)	6.5 (6.1, 6.9)	0.064
Alb (g/dL)	4.2 (3.8, 4.3)	4.0 (3.7,4.4)	0.689
AST (IU/L)	9.5 (9.0, 20.0)	11.0 (6.5, 14.0)	0.445
ALT (IU/L)	12.0 (9.3, 17.5)	9.0 (8.0, 12.5)	0.094
LDH (IU/L)	193.5 (182.8, 213.5)	171.0 (148.5, 194.5)	0.076
ALP (IU/L)	318.0 (193.8, 348.8)	168.0 (157.5, 241.5)	0.039
BUN (mg/dL)	61.0 (56.3, 72.3)	60.0 (50.5, 68.5)	0.405
Cr (mg/dL)	11.8 (9.7, 13.4)	12.2 (10.2, 13.8)	0.799
UA (mg/dL)	7.6 (5.4, 8.9)	7.6 (6.5, 8.0)	0.744
β2-MG (mg/L)	21.7 (18.2, 22.9)	21.0 (20.0, 24.5)	0.799
NT-proBNP (pg/mL)	3323 (1473, 6494)	2657 (826, 12645)	0.638
Ca (mg/dL)	8.8 (8.5, 9.6)	9.1 (8.9, 9.5)	0.611
P (mg/dL)	5.9 (5.0, 6.9)	5.4 (4.9, 6.2)	0.537
iPTH (pg/mL)	152.5 (70.8, 259.8)	174.0 (82.0, 223.0)	0.914
Fe (μg/dL)	64.5 (55.5, 82.8)	73.0 (50.0, 94.0)	0.612
Ferritin (μg/L)	74.5 (30.3, 241.8)	245.0 (131.0, 393.5)	0.120
hs-CRP (mg/dL)	0.10 (0.06, 0.31)	0.06 (0.03, 0.13)	0.403
TG (mg/dL)	129.0 (91.3, 190.0)	100.0 (77.0, 141.0)	0.277
TC (mg/dL)	152.0 (123.3, 184.0)	163.0 (145.5, 201.0)	0.246
LDL-C (mg/dL)	79.5 (54.5, 94.0)	93.0 (72.5, 123.5)	0.246
HDL-C (mg/dL)	39.0 (27.5, 54.5)	42.0 (39.0, 51.5)	0.663
BS (mg/dL)	148.0 (98.3, 195.5)	107.0 (96.5, 116.5)	0.180
HbA1c (%)	7.0 (6.6, 7.9)	5.2 (4.8, 5.3)	0.0002
Hematologic data			
WBC (10^3^/μL)	5.55 (4.90, 6.25)	5.10 (4.15, 5.85)	0.310
Hb (g/dL)	11.8 (11.0, 12.6)	11.0 (10.5, 12.1)	0.232
Ht (%)	37.0 (34.0, 38.9)	34.1 (33.2, 36.2)	0.168
PLt (10^4^/μL)	19.1 (17.4, 23.6)	15.0 (10.6, 17.9)	0.020
Dialysis parameters			
HD duaration (y)	5.2 (2.4, 6.3)	4.1 (1.1, 13.7)	0.971
Kt/V_urea_	1.32 (1.25, 1.42)	1.31 (1.21, 1.45)	0.968

Data are presented in number (%) or median (interquartile range).

*p-values were calculated by Mann-Whitney test for continuous variables or Fishers exact test for categorical variables.

*Abbreviations: DM* diabetes mellitus, *TP* total protein, *Alb* albumin, *AST* aspartate aminotransferase, *LDH* lactase dehydrogenase, *ALP* alkaline phosphatase, *BUN* blood urea nitrogen, *Cr* reatinine, *UA* urinary acid, *β2-MG* beta2-microglobulin, *NT-proBNP* N-terminal pro-brain natriuretic peptide, *Ca* calcium, *P* phosphorus, *iPTH* intact parathyroid hormone, *Fe* ferrum, *hsCRP* high-sensitivity C-reactive protein, *TG* triglyceride, *TC* total cholesterol, *LDL-C* low-density lipoprotein cholesterol, *HDL-C* high-density lipoprotein cholesterol, *BS* blood sugar, *HbA1c* hemoglogin A1c, *WBC* white blodd cell, *Hb* hemoglobin, *Ht* hematocrit, *PLt* platelet, *HD* hemodialysis, *Kt/Vurea* Kt/V for urea.

### Quantitative real-time polymerase chain reaction (qRT-PCR) assay

Periodontopathogens were identified using a quantitative real-time polymerase chain reaction (qRT-PCR) [12] based on 16S rRNA genes. Bacteria-specific primer pairs based on the species-specific region on the 16S rRNA genes are shown in Table 2[13,14]. Each 50 μL PCR reaction mixture contained 5 μL of the sample, 5 μL of 10 x PCR buffer (TaKaRa, Shiga, Japan), 1.25 units of TaqDNApolymerase (TaKaRa), 0.2 mM of each deoxyribonucleotide (TaKaRa), 1.0 mM of each primer, and 1.0 mM MgCl_2_ for *Aggregatibacter actinomycetemcomitans* (*A. actinomycetemcomitans*) or 1.5 mM MgCl_2_ for *Porphyromonas gingivalis* (*P. gingivalis*). PCR amplification was performed in a DNA thermal cycler (PTC-200, MJ Research, Boston, MA). The temperature profile for *A. actinomycetemcomitans* and *Prevotella intermedia* (*P. intermedia*) included an initial step at 95°C for 2 minutes followed by 36 cycles of 94°C for 30 seconds, 55°C for 1 minute, 72°C for 2 minutes, and a final step at 72°C for 10 minutes. The PCR temperature profile for *P. gingivalis* included an initial step at 95°C for 2 minutes followed by 36 cycles of 95°C for 30 seconds, 60°C for 1 minute, 72°C for 1 minute, and a final step at 72°C for 2 minutes.

**Table 2:** Species-specific and ubiquitous primers for PCR

**Species**	**Primers pairs (5’ to 3’)**	**Amplicon size in bp**
*Aggregatibacter antinomycetemcomitans*	AAACCCATCTCTGAGTTCTTCTTC	557
	ATGCCAACTTGACGTTAAT	
*Porphyromonas gingivalis*	ACTGTTAGCAACTACCGATGT	404
	AGGCAGCTTGCCATACTGCG	
*Prevotella intermedia*	TCAACATCTCTGTATCCTGCGT	575
	TTTGTTGGGGAGTAAAGCGGG	

*Abbreviations: PCR* polymerase chain reaction.

### Serum IgG antibody titer measurement

Specific serum IgG titers were measured by ELISA using sonicated whole cell extracts of each periodontopathogen. Briefly, the microtiter plates were coated with sonicated whole cell extracts of *P. gingivalis* ATCC 33277, *A. actinomycetemcomitans* ATCC 33384 and *P. intermedia* ATCC 25611. The 96-well microtiterplates (EIA plate, Costar, Cambridge, MA) were coated with sonicated extracts (10 μg/mL) in a carbonate buffer, and incubated for 2 hours at 37°C. After blocking with 2% BSA in carbonate buffer, the plates were washed 3 times with PBS-T (1 × PBS, 0.05% Tween 20, pH 7.2). Serially diluted reference positive control serum (25 to 214, 100 μL per well) and single diluted (210 for *P. gingivalis* and *A. actinomycetemcomitans*, and 28 for *P. intermedia*) patient serum were added into each well in duplicate and the plates were incubated for 2 hours at 37°C. Following incubation, the plates were washed again 3 times. Subsequently, 100 μL per well of alkaline phosphatase-conjugated goat anti-human IgG (Sigma Chemical Co., USA) was added. Following incubation, the plates were washed 3 times and developed with phosphate substrate (Sigma104). The optical density was read using a Microplate Reader (SOFT MaxTM) at 405 nm with a 650 nm reference wavelength. Antibody titer was calculated according the method of Wang *et al*. [15].

### Magnetic resonance imaging (MRI) evaluation of the brain

All subjects received an MRI of the brain. The slice thickness was 5 mm with an interslice gap of 1 mm. Criteria of cerebral infarction was defined as a low-intensity area on the T1-weighted image and as a high-intensity area on the T2-weighted image [16]. Cerebral infarction included both symptomatic and silent cerebral infarction. We defined silent cerebral infarction as a focal area ≧3 mm and <20 mm in diameter in both T1- and T2- weighted scans, while dilated Virchow-Robin spaces were excluded. Cerebral infarction history was determined by checking the medical records of all subjects, independent of their MRI outcome. We defined silent cerebral infarction as evidence on MRI of one or more infarctions, without a history of a stroke.

### Statistical analysis

Baseline characteristics of the study populations were presented in number (%) for categorical variables or median (interquartile range) for continuous variables. Differences in continuous and categorical variables were examined with Mann–Whitney test and Fishers exact test for two group comparisons, respectively. We evaluated statistical correlations between cause of ESRD (the diabetic and non-diabetic groups) and periodontal parameters and serum IgG titers specific to them using Mann–Whitney test. We used Fishers exact test to evaluate statistical correlations between cause of ESRD and cerebral infarction. We analyzed statistical correlations between cause of ESRD and 3 periodontopathogens in saliva and subgingival plaque using both Mann–Whitney and Fishers exact test. The alpha level was set at 0.05. All statistical analyses were performed with the aid of statistical software (SPSS Statistics®, Version 20, IBM).

## Results

### Characteristics of the study population

Seventeen patients of 149 HD patients in Saku Central Hospital were excluded from the study because they had at least one of the following exclusion criteria; (i) known systemic diseases, (ii) history and/or presence of other infections, or (iii) systemic antibiotic, immunosuppressive or periodontal treatment in 6 months prior to the sample collection. Only 21 of 132 HD patients consented to this study. These patients (16 males, 5 females) with a median duration of 4.7 years of HD therapy (from 0.3 to 27.6 years) were enrolled for analysis. The demographic characteristics of the study population are presented in Table 1. Patient age ranged from 40 to 86 years (median 64.0 years). Primary renal diseases of the study population were as follows: diabetic nephropathy (38%), chronic glomerulonephritis (14%), and hypertensive glomerulosclerosis (10%), and unknown (38%). There were 8 HD patients with diabetic nephropathy (2 females and 6 males, median age 63.0 years) and 13 with non-diabetic nephropathy (3 females and 10 males, median age 64.0 years). The 21 HD patients included 8 with diabetic nephropathy and 13 with non-diabetic nephropathy. The majority of HD patients were undergoing 4 hours of HD 3 times/week. HD was prescribed in these patients with single-use hollow-fiber dialyzers equipped with polysulfone or polymethylmethacrylate membranse. The dialysate used was a standard ionic composition and bicarbonate-based buffer.

### Periodontal evaluation

**Figure 1:**
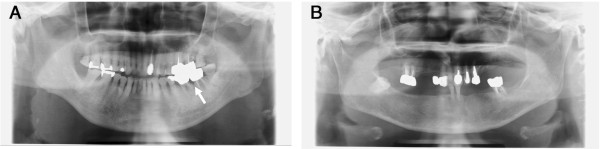
**Periapical radiogram.** Representative periapical radiogram findings of non-diabetic **(A)** and diabetic **(B)** nephropathy patients are shown. Mild horizontal and vertical alveolar bone resorption was found in a non-diabetic **(A)** nephropathy patient. Moderate horizontal alveolar bone resorption and a lot of missing teeth were found in a diabetic **(B)** nephropathy patient. An arrow indicates vertical alveolar bone resorption.

Subjects who had at least 1 site with a tooth pocket depth of ≥ 4 mm and/or showed bone loss on the radiograms were considered to have periodontitis. In the present study, most subjects had periodontitis. Representative periapical radiogram findings of HD patients are presented in Figure 1. We evaluated the statistical correlations between cause of ESRD and clinical oral and radiographic parameters. The number of missing teeth, % of sites with PPD ≥ 4 mm, % of sites exhibiting BOP, and % of sites with bone loss ≥ 25% on radiograms also tended to be higher among patients in the diabetic nephropathy group than in the non-diabetic nephropathy group. However, the differences were not statistically significant (Table 3).

**Table 3:** Clinical oral and radiographic data of the study populations

	**DM (n=8)**	**Non-DM (n=13)**	P value*
Number of missing teeth	3.0 (1.0, 12.3)	2.0 (0, 4.0)	0.340
% of sites with PPD ≥ 4 mm	3.3 (0.6, 6.0)	3.1 (1.2, 5.1)	0.942
% of sites with CAL ≥ 4 mm	15.2 (12.9, 22.4)	9.1 (4.2, 33.5)	0.158
% of sites exhibiting BOP	30.1 (1.7, 47.1)	14.0 (7.8, 33.0 )	0.717
% of sites with bone loss ≥ 25	18.7 (5.8, 45.0)	4.2 (3.7, 16.0)	0.137
% on radiograms			

Data are presented in median (interquartile range).

*p-values were calculated by Mann-Whitney test.

*Abbreviations: DM* diabetes mellitus, *PPD* probing pocket depth, *CAL* clinical attachment level, *BOP* bleeding on probing.

### Microbiological evaluation

**Figure 2:**
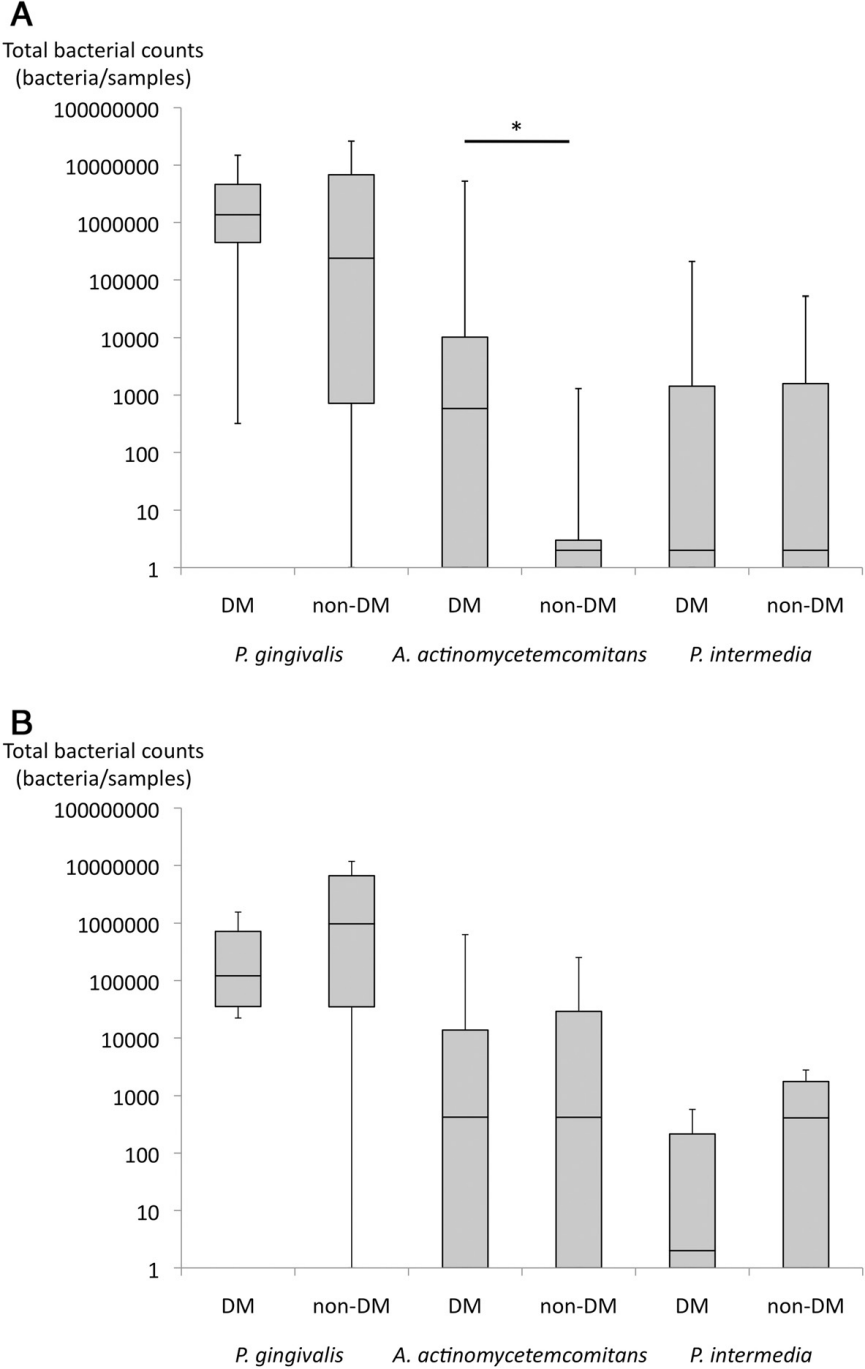
**PCR analysis.** The results of PCR analysis of subgingival plaque **(A)** and saliva **(B)** are demonstrated. An asterisk (*) indicates a significant difference. A boxplot shows median, and interquatile range between 25th and 75th percent. Data are presented in number (%) or median (interquartile range). P-values were calculated by Mann–Whitney test.

The results of PCR analysis on subgingival plaque and saliva samples for all subjects are shown in Figure 2 and Tables 4 and 5. Although each pathogen did not demonstrate the statistical differences between the two groups, *A. actinomycetemcomitans* in the diabetic nephropathy group tended to have a higher prevalence rate compared to the non-diabetic nephropathy group in both saliva and plaque. Moreover, the patients with diabetic nephropathy had significantly more *A. actinomycetemcomitans* quantitatively compared to the patients with non-diabetic nephropathy (P = 0.038) in dental plaque. *P. gingivalis* and *P. intermedia* in both dental plaque and saliva did not differ quantitatively between the diabetic and non-diabetic groups. There were 5 (56%) and 3 (33%) patients who were positive for *A. actinomycetemcomitans* salivary and subgingivally of the 9 patients with cerebral infarction, respectively.

**Table 4:** Number (%) of patients who were positive for each pathogen in saliva

	***A. actinomycetemcomitans***	***P. intermedia***	***P. gingivalis***
DM (n=8)	4 (50)	2 (25)	7 (88)
Non-DM (n=13)	5 (38)	6 (46)	9 (69)

*Abbreviations: DM* diabetes mellitus.

**Table 5:** Number (%) of patients who were positive for each pathogen in subgingival plaque

	***A. actinomycetemcomitans***	***P. intermedia***	***P. gingivalis***
DM (n=8)	5 (63)	3 (38)	8 (100)
Non-DM (n=13)	3 (23)	5 (38)	10 (77)

*Abbreviations: DM* daibetes mellitus.

### Serum IgG titers to periodontopathogens

**Figure 3:**
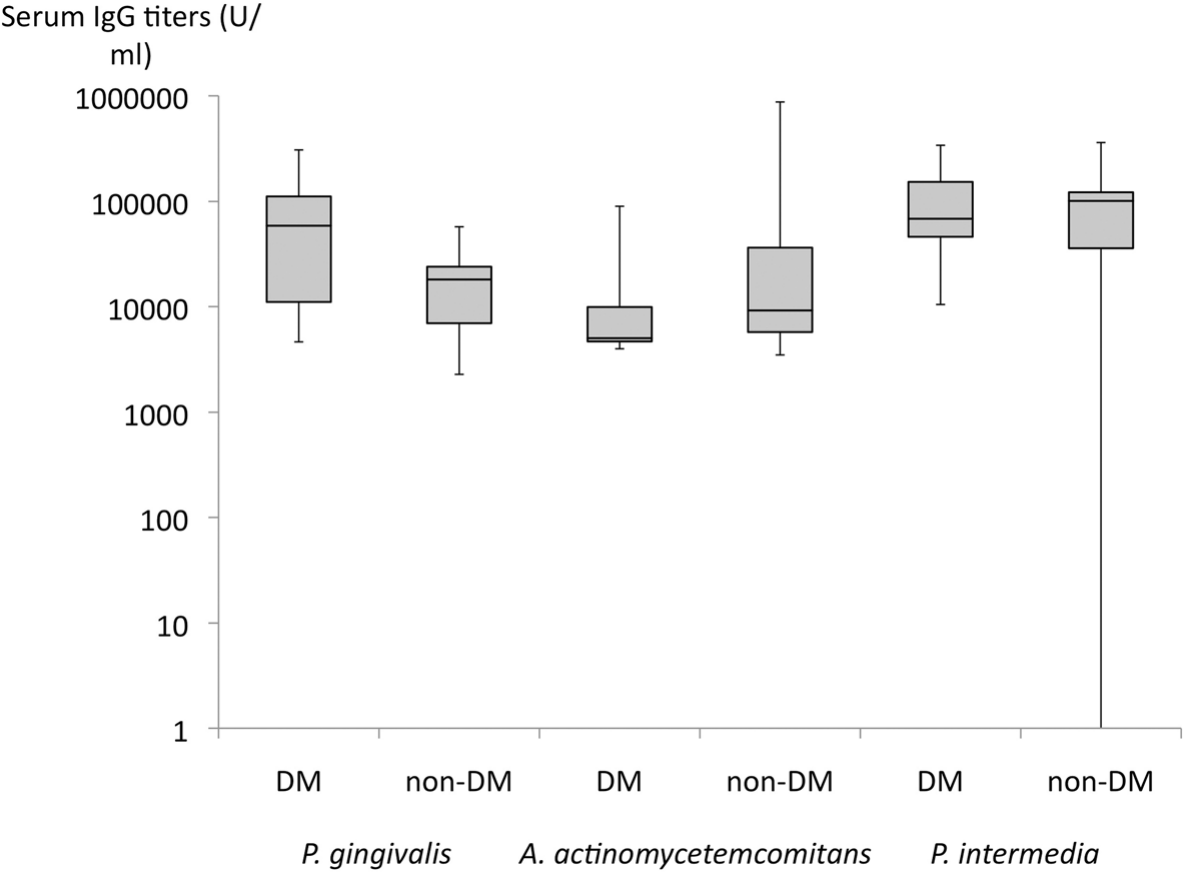
**ELISA analysis.** The results of serum IgG antibody titers specific to the 3 periodontopathogens measured by ELISA analysis are demonstrated.

Serum IgG titers specific to the 3 periodontopathogens are shown in Figure 3. No statistical difference was found in the IgG titers between the diabetic and non-diabetic groups.

The median serum anti-*A. actinomycetemcomitans* antibody levels were 90026 in the 5 patients who were salivary positive for it, while the levels were 4891 in the 3 patients who were subgingivally positive for it.

### Brain MRI evaluation

**Figure 4:**
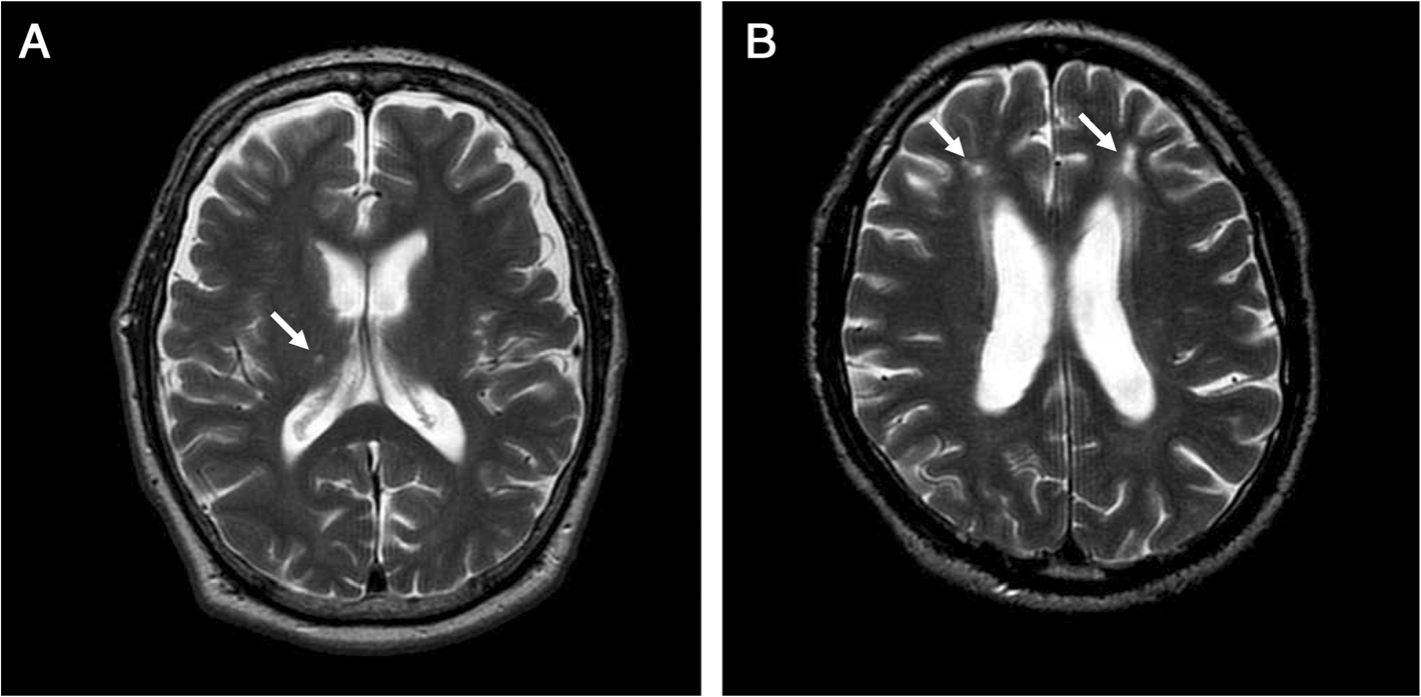
**Brain MRI.** Representative brain MRI findings (T2-WI) of non-diabetic **(A)** and diabetic **(B)** nephropathy patients are shown. Arrows indicate cerebral infarctions.

Representative brain MRI findings (T2-WI) of HD patients are presented in Figure 4. It was found that 75% of the patients with diabetic nephropathy (6/8) had cerebral infarction, whereas 23% of those with non-diabetic nephropathy (3/13) had cerebral infarction (P = 0.029). Among patients with cerebral infarction, all showed lacunar infarction. Eight patients had silent cerebral infarction and only one patient with diabetic nephropathy had symptomatic cerebral infarction.

## Discussion

The present study demonstrated the periodontal and brain status of 21 HD patients. Our results showed that patients with diabetic nephropathy had significantly more *A. actinomycetemcomitans* compared to patients with non-diabetic nephropathy in dental plaque. We also found that the patients with diabetic nephropathy showed a significantly higher incidence of cerebral infarction compared to those with non-diabetic nephropathy.

Kshirsagar *et al.* reported a retrospective HD cohort in which moderate to severe periodontal disease was associated with a 5-fold increase in cardiovascular mortality after 18 months of follow-up [17]. Furthermore, a large-scale systemic review that included 8 case-controlled and 18 cross-sectional reports suggested that periodontal disease may be associated modestly with atherosclerosis, myocardial infarction, and cerebrovascular disease [18]. The cardiovascular risk seemed to be highest among those who showed both evidence of some chronic, low-grade infection, and elevated CRP levels [19].

Takeuchi *et al*. evaluated the composition of subgingival microbiota in 81 patients with CKD with that in 62 healthy individuals by performing PCR with gingival crevicular fluid [20]. They found that *Tannerella forsythia* (*T. forsythia*), *Treponema denticola* (*T. denticola*), *Prevotella nigrescens*, and *Candida albicans* were more frequent in patients with CKD than in controls. Bastos *et al*. showed that red bacterial complex (*P. gingivalis*, *T forsythia*, and *T. denticola*) were more frequent in patients with chronic periodontitis and CKD than in healthy individuals [21]. Wara-aswapati *et al*. also suggested that red complex bacteria were predominant periodontal pathogens of the moderate to severe form of chronic periodontitis in a Thai population [22]. The presence of red bacterial complex was associated with the severity of disease. Therefore, these pathogens should be considered as targets for the prevention and treatment of periodontal disease.

It is commonly known that individuals with diabetes mellitus (DM) are at risk of periodontitis [23]. The high prevalence of periodontitis among diabetic patients is mainly due to their high susceptibility to infection. The implications for oral health and the provision of dental care for people with DM are significant, because numerous studies have demonstrated an association between DM and periodontitis [24]. Furthermore, DM patients with periodontal disease have an increased risk of severe systemic disease compared to those without periodontal disease [25]. A recent meta-analysis of the efficacy of periodontal treatment on glycemic control in diabetic patients suggested that such treatment could lead to significant reductions of glycated hemoglobin [26].

Ghizoni *et al.* reported that stroke patients had deeper pockets, more severe attachment loss, increased BOP, increased plaque index, and in their pockets harbored increased levels of *P. gingivalis*[27]. These findings suggest that periodontal disease is a risk factor for the development of cerebral hemorrhage or infarction.

*A. actinomycetemcomitans* is a gram-negative, facultatively anaerobic coccobaccillus and is considered to be the major etiologic agent of localized aggressive periodontitis [28]. It also contributes to chronic periodontitis. Studies in Chinese, Korean, and southern Thai populations have shown high detection frequencies of *A. actinomycetemcomitans* ranging approximately from 40% to 75% of the sampling sites [29-32]. In contrast, a study in a Japanese population reported that less than 10% of the chronic periodontitis diseased site was positive for *A. actinomycetemcomitans*[33]. Furthermore, this microorganism has been isolated from several other non-oral infections, including endocarditis [34] and pericarditis [35]. Recently, these species were identified in atheromatous plaques of cardiovascular disease patients [36], suggesting a possible role for *P. gingivalis* and *A. actinomycetemcomitans* in the development of this lesion.

These results are supported by recent studies showing that elevated serum anti-*A. actinomycetemcomitans* antibody levels predicts stroke [37] and coronary heart disease [38]. *A. actinomycetemcomitans* possesses a number of putative virulence factors, including a leukotoxin that targets and destroys specific host immune cells. Leukotoxin is also involved in the adhesion of *A. actinomycetemcomitans*[39]. The organism takes advantage of its high adhesiveness and is capable of rapid invasion and spread through eukaryotic cells [40]. Ten *et al.* reported that periodontitis patients infected with *A. actinomycetemcomitans* harbored *A. actinomycetemcomitans*-specific T-cells in peripheral blood, and T-cells expressed RANK ligand (RANKL) in response to *A. actinomycetemcomitans*[41]. RANKL from T-cells stimulates vascular smooth muscle cells to produce matrixmetalloproteinase-9. These cells destabilize atherosclerotic plaque and were reported to be elevated in coronary artery plaque in patients with acute myocardial infarction [42].

In the present study, we selected *A. actinomycetemcomitans, P. intermedia,* and *P. gingivalis* for analysis after the previous study, which reported an association between serum anti-periodontal pathogen antibody and ischemic stroke. Hosomi *et al.* reported that anti-*P. intermedia* antibody was associated with carotid artery atherosclerosis, and that anti-*P. intermedia* antibody may be associated with atherothrombotic stroke through its association with carotid artery atherosclerosis [43]. It was found that patients with diabetic nephropathy had significantly more *A. actinomycetemcomitans* and cerebral infarction compared to patients with non-diabetic nephropathy in dental plaque. The results of degree of periodontitis also tended to be higher among patients in the diabetic nephropathy group, although the difference was not significant. Therefore, we thought that *A. actinomycetemcomitans* may play a role, at least a part, in the development of cerebral infarction in Japanese HD patients with diabetic nephrology. However, some of the results were not consistent with our hypothesis. Increased systemic inflammation as a pathogenic link was not supported by serum hs-CRP levels and the IgG titers against *A. actinomycetemcomitans*. Because periodontal *A. actinomycetemcomitans* infection is generally a local burden, it may not influence systemic inflammatory reaction until periodontitis becomes more advanced. At this point, we cannot clarify the discrepancy between *A. actinomycetemcomitans* infection and hs-CRP levels. Further investigation is required.

The virulence of *A. actinomycetemcomitans* is still not well understood, but it is able to produce a heat-labile leukotoxin, which belongs to the repeat-in-toxin (RTX) family. The gene ltxA encodes a structural leukotoxin and genes ltxB and ltxD encode proteins required for its secretion. Gene ltxC encodes an acyltransferase that is responsible for the modification of proto-toxin to the active toxin [44]. Moreover, the leukotoxin production has been associated with evasion against the defense cells of the periodontal tissues [45,46]. In this study, we cannot reveal the virulence mechanisms among the dialysis, DM, and cerebral infarction. Thus, further investigation is needed to clarify the pathological mechanisms.

We have some limitations in this study. Firstly, it is difficult to reveal a causal relationship among periodontal *A. actinomycetemcomitans* infection, diabetic nephropathy, and cerebral infarction, because this is a cross-sectional observation study. Secondly, some statistical analyses were incomplete because the sample size was too small to calculate. Thus, further investigation is required.

## Conclusions

Periodontal pathogens, particularly *A. actinomycetemcomitans*, may play a role, at least a part, in the development of cerebral infarction in Japanese HD patients with diabetic nephropathy.

## Competing interests

The authors declare that they have no competing interests.

## Authors’ contributions

MM performed the statistical analysis and drafted the manuscript. JS conceived of the study, and participated in its design and coordination and helped to draft the manuscript. SY participated in its design and coordination. RM performed periodontal examination. NA and NA carried out the microbiological evaluation and immunoassays. MI, NK, HA, IK, YI, and MI contributed to the discussion and reviewed and edited the manuscript. All authors read and approved the final manuscript.

## Pre-publication history

The pre-publication history for this paper can be accessed here:

http://www.biomedcentral.com/1471-2334/13/557/prepub
